# Supplementary material for: Splice-Junction-Based Mapping of Alternative Isoforms in the Human Proteome
Source: Cell Rep. Author manuscript; Available in PMC 2020 Jan 15. (PMC6961840; doi:10.1016/j.celrep.2019.11.026)
Supplement: 2 [file NIHMS1546469-supplement-2.zip › DF1/PXD010154/Colon/Colon_14_AFMID_QQTLCQGEWK.pdf]

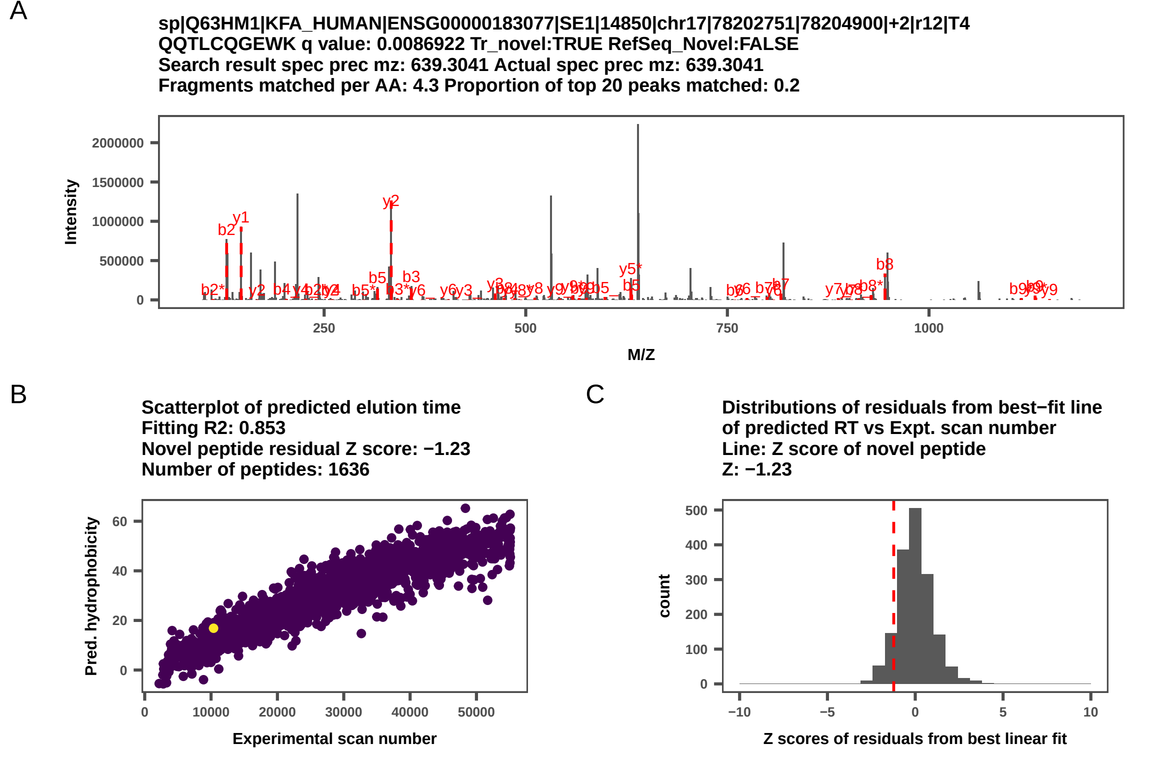
A
sp|Q63HM1|KFA_HUMAN|ENSG00000183077|SE1|14850|chr17|78202751|78204900|+2|r12|T4
QQTLCQGEWK q value: 0.0086922 Tr_novel:TRUE RefSeq_Novel:FALSE
Search result spec prec mz: 639.3041 Actual spec prec mz: 639.3041
Fragments matched per AA: 4.3 Proportion of top 20 peaks matched: 0.2
Intensity
2000000
1500000
1000000
500000
0
y1
b2
y2
b5
b3
y5*
b8
b2*
b4
b5*
b3*
y6
b5
b8*
250
500
750
1000
M/Z
B
Scatterplot of predicted elution time
Fitting R2: 0.853
Novel peptide residual Z score: −1.23
Number of peptides: 1636
Pred. hydrophobicity
60
40
20
0
0
10000
20000
30000
40000
50000
Experimental scan number
C
Distributions of residuals from best−fit line
of predicted RT vs Expt. scan number
Line: Z score of novel peptide
Z: −1.23
count
500
400
300
200
100
0
−10
−5
0
5
10
Z scores of residuals from best linear fit
